# βarrestin-1 regulates DNA repair by acting as an E3-ubiquitin ligase adaptor for 53BP1

**DOI:** 10.1038/s41418-019-0406-6

**Published:** 2019-09-10

**Authors:** Ainhoa Nieto, Makoto R. Hara, Victor Quereda, Wayne Grant, Vanessa Saunders, Kunhong Xiao, Patricia H. McDonald, Derek R. Duckett

**Affiliations:** 1 Department of Cancer Physiology, Moffitt Cancer Center, Tampa, FL 33612 USA; 2 Department of Medicine, Duke University Medical Center, Durham, NC 27710 USA; 3 Department of Drug Discovery, Moffitt Cancer Center, Tampa, FL 33612 USA; 4 Department of Molecular Medicine, The Scripps Research Institute, Jupiter, FL 33458 USA; 5 Present Address: Novartis Institutes for Biomedical Research, Cambridge, MA 02139 USA

**Keywords:** DNA-binding proteins, Tumour-suppressor proteins, Gene expression

## Abstract

Cellular DNA is constantly under threat from internal and external insults, consequently multiple pathways have evolved to maintain chromosomal fidelity. Our previous studies revealed that chronic stress, mediated by continuous stimulation of the β_2_-adrenergic-βarrestin-1 signaling axis suppresses activity of the tumor suppressor p53 and impairs genomic integrity. In this pathway, βarrestin-1 (βarr1) acts as a molecular scaffold to promote the binding and degradation of p53 by the E3-ubiquitin ligase, MDM2. We sought to determine whether βarr1 plays additional roles in the repair of DNA damage. Here we demonstrate that in mice βarr1 interacts with p53-binding protein 1 (53BP1) with major consequences for the repair of DNA double-strand breaks. 53BP1 is a principle component of the DNA damage response, and when recruited to the site of double-strand breaks in DNA, 53BP1 plays an important role coordinating repair of these toxic lesions. Here, we report that βarr1 directs 53BP1 degradation by acting as a scaffold for the E3-ubiquitin ligase Rad18. Consequently, knockdown of βarr1 stabilizes 53BP1 augmenting the number of 53BP1 DNA damage repair foci following exposure to ionizing radiation. Accordingly, βarr1 loss leads to a marked increase in irradiation resistance both in cells and in vivo. Thus, βarr1 is an important regulator of double strand break repair, and disruption of the βarr1/53BP1 interaction offers an attractive strategy to protect cells against high levels of exposure to ionizing radiation.

## Introduction

Mammalian cells are continuously bombarded by DNA damaging insults which threaten cell viability and genomic integrity [1]. Amongst the most toxic lesions are DNA double-strand breaks (DSBs), as failure to properly repair even a single DSB has dire consequences triggering cell death, cell cycle arrest, or gross chromosomal rearrangements such as deletions, translocations, and amplifications; all causal drivers of tumorigenesis [2]. Breaks in both strands of DNA can be induced by numerous sources such as, the collapse of replication forks, reactive products of oxidative metabolism, as well as from external sources such as, ionizing radiation (IR). To repair DSBs cells utilize two major pathways namely, nonhomologous end joining (NHEJ), which occurs throughout the cell cycle and directly rejoins the broken ends with minimal processing [3], and homologous recombination (HR), which requires large stretches of sequence homology necessary for repairing lesions with high fidelity. Typically, HR requires the sister chromatid to serve as a template and thus is restricted to S/G2 phases of the cell cycle [4]. Coordination of these repair processes is essential, and tightly regulated to ensure that the appropriate counter is taken to ameliorate the threat to the cell [5].

The tumor suppressor protein p53-binding protein 1 (53BP1) is an important modulator of DSB repair [6]. This is exemplified in mice deficient in 53BP1, which are sensitive to IR and have significant defects in adaptive immune response due to defective repair of programmed DSBs such as, class switch recombination and V(D)J recombination in lymphocytes [7, 8]. Furthermore, 53BP1 knockout mice are growth retarded and tumor prone [9, 10]. 53BP1 plays opposing roles to BRCA1 (breast cancer susceptibility gene-1) during the initial stages of DSB repair by inhibiting accumulation of BRCA1 at the break site and prevents degradation of DNA at stalled replication forks [11–15]. This is particularly revealing in the setting of BRCA1 deficiency where HR is markedly reduced and DSBs that occur during S phase are funneled through 53BP1 for repair by NHEJ. Accordingly, silencing 53BP1 rescues the chromosomal instability, embryonic lethality and tumorigenicity in brca1^−/−^ deficient mice [16–18].

The molecular events mediated by 53BP1, its recruitment to sites of DNA damage, and its interactions with other proteins are tightly controlled [19, 20]. Here, we report a novel mechanism of 53BP1 regulation. We show that βarrestin-1 (βarr1), originally identified as a molecule that desensitizes G protein-coupled receptor signaling but now known to also have roles in regulating MAPK, PI3K/AKT, and c-Src signaling [21, 22], plays an essential role in controlling the levels of basal and DNA damage-inducible 53BP1. Akin to its described function as an MDM2 ubiquitin ligase adaptor for controlling p53 destruction [23], we now show that βarr1 functions as an E3-ubiquitin ligase adaptor that controls 53BP1 levels and thus influences the mode of repair of DNA DSBs. Importantly, our studies establish that disabling βarr1-directed 53BP1 degradation confers remarkable resistance to IR, both in vitro and in vivo, underscoring the physiological roles of βarr1 as a mediator of the DNA damage response (DDR).

## Materials and methods

### Reagents

Unless otherwise noted, chemicals were purchased from Sigma Aldrich. Purified Rad18 and Rad6 proteins were purchased from Abcam and synthetic siRNAs were obtained from Dharmacon RNA Technologies. The antibodies used in this study were obtained from the following sources: 53BP1 (Santa Cruz; Abcam); ubiquitin (Santa Cruz, K4D1); βarr1 (Cell Signaling); βarr2 (Abcam); LDH (BD Biosciences); α-tubulin (Cell Signaling); γ-H2AX (Cell Signaling; Millipore); Anti-mouse IgG-HRP and anti-rabbit IgG-HRP (GE Healthcare); Alexa Fluor 647 Donkey anti-rabbit IgG (Invitrogen); Alexa Fluor 555 Donkey anti-rabbit IgG (Invitrogen); Alexa Fluor 488 Donkey anti-mouse IgG (Invitrogen). Rabbit polyclonal βarr1 antibody (A1CT) was generated as previously described [24].

### Cell culture conditions and treatments

Wild-type mouse embryo fibroblasts (MEFs), βarr1^−/−^ MEFs, and βarr2^−/−^ MEFs were prepared according to the 3T3 protocol [25, 26]. Established MEFs were maintained in Dulbecco’s Modified Eagle Medium with 10% FBS and 2 mM l-glutamine at 37 °C with a 5% CO_2_ atmosphere in a humidified incubator. U2OS and HEK293 cells were maintained in Modified Eagle Medium with 10% FBS and 2 mM l-glutamine with the same conditions above. For knockdown of βarr1 and Rad18, cells were transfected with GeneSilencer (Gene Therapy Systems, San Diego) as described [27]. Two different types of control siRNA were used in this study: CTL1: siGLO control RNAi (catalog #: D-001620-02) from Thermo Scientific and CTL2 (5′-UUCUCCGAACGUGUCACGU-3′) [28]. siRNA for Rad18 was from Thermo Scientific (SMARTpool: ON-TARGETplus RAD18 siRNA). Lentiviral pLKO1 constructs containing shRNA sequences directed against βarr1 (CCGGGCCAGTAGATACCAATCTCATCTCGAGATGAGATTGGTATCTACTGGCTTTTTG), 53BP1 (CCGGCGCGTCATCACAGATGTTTATCTCGAGATAAACATCTGTGATGACGCGTTTTTG), Rad18 (GGTTAACATTCCAGAAAGTCA) and CTL (CCGGCAACAAGATGAAGAGCACCAACTCGAGTTGGTGCTCTTCATCTTGTTGTTTTT) were purchased from Sigma Aldrich. Retroviral N-Myc-53BP1 WT pLPC-Puro construct for 53BP1 overexpression was purchased from Addgene (#19836). Lentiviral CRISPR-Cas9 construct containing sgRNA against βarr1(CATCGACCTCGTGGACCCTG).

### Co-immunoprecipitation

Immunoprecipitation analyses were performed as described [29]. Input samples were run with 5% of the IP lysate. To detect polyubiquitylation of 53BP1, 20 μM MG132 was added to plates 4 h prior to harvesting cells, and 10 mM N-ethylmaleimide and 20 μM MG132 were added to the lysis buffer (50 mM Tris pH 7.4, 150 mM NaCl, 0.1% Chaps, 0.1 mg/ml BSA, 1 mM PMSF, 1 mM EDTA, with Halt protease and phosphatase inhibitor cocktail (Pierce)).

### Immunoblotting

Western blot analyses were conducted as previously described [29]. Briefly, SDS-PAGE gel electrophoresis was performed using NuPAGE 4–12% Bis-Tris gels (Invitrogen) and transferred to nitrocellulose membranes by semidry transfer, using trans-blot transfer medium (Bio-Rad). Blots were blocked with blocking buffer (5% skimmed milk in PBS with 0.02% Tween-20) then incubated at 4 °C overnight with primary antibodies, diluted in blocking buffer. Blots were washed three times in PBS with 0.02% Tween-20, and then incubated with secondary antibodies diluted in blocking buffer for 1 h at room temperature. Blots were then washed three times in PBS with 0.02% Tween-20, and developed by SuperSignal West Pico/Femto solution (Pierce). Immunoblots were quantified by densitometry using the ImageJ [30]. Alternatively, membranes were blocked in Odyssey blocking buffer (LI-COR Biosciences) and incubated overnight at 4 °C with primary antibodies. After repeated washes with TBS-T [20 mM tris (pH 7.6), 140 mM NaCl, and 0.1% Tween-20], blots were incubated with the appropriate IRDye-conjugated secondary antibody (LI-COR Biosciences) and imaged using the LI-COR Odyssey. Bands were quantified using the Odyssey software (LI-COR Biosciences).

### Immunofluorescence analyses

MEF and U2OS cells were grown on eight-well chamber slides (Labtek), coverslips or 96-well Viewplates (Perkin Elmer) and exposed to increasing levels of γ-irradiation. Cells were allowed to recover for the indicated times, washed in PBS, fixed with 4% paraformaldehyde for 15 min at room temperature, permeabilized with 0.1% Triton X-100/PBS, and blocked for 30 min in blocking buffer (1% goat serum, 0.3% BSA in PBS). Cells were incubated overnight at room temperature in primary antibody diluted as per manufacturer’s recommendation in blocking buffer overnight at 4 °C, washed three times with 0.05% Tween-20/PBS, incubated with Alexa Fluor 647 Donkey anti-rabbit IgG, Alexa Fluor 555 Donkey anti-rabbit IgG, and/or Alexa Fluor 488 Donkey anti-mouse IgG secondary antibody diluted 1:1000 in blocking buffer for 1 h at room temperature. Nuclei were stained for 20 min at room temperature with 4,6-diamidino-2-phenylindole (Sigma/AppliChem) in PBS, diluted at 1:5000 from a 1 mg/ml stock. MEF cells were analyzed by mounting coverslips on microscope slides in SlowFade (Molecular Probes) and viewed with an Olympus IX81 Fluoview FV1000 confocal microscope fitted with a 100× objective. To quantify 53BP1 foci, 96-well samples were imaged with an automated high-content Incell 1000 microscope (GE Healthcare) using a 20× Nikon objective and analyzed with the Incell Developer Toolbox version 1.6 software. U2OS cells were imaged using a Leica SP8 Laser Scanning Confocal Microscope fitted with a 60× objective to view the cells and the Cytation 5 cell imaging multimode reader (Biotek) was used for imaging and quantitative analysis. We determined the frequency of nuclei with more than five 53BP1 foci. At least 100 nuclei were analyzed for each sample.

### Clonogenic cell survival assay

MEF cells (500) were plated in six-well dishes (*n* = 6) and irradiated with graded doses (0, 1, 2, 4, or 8 Gy) of γ-radiation using a 137Cs source (Gammacell 40 irradiator; 3.7 Gy/min). After 7–10 days of cell growth, during which media was changed every 2–3 days, cell colonies were fixed with 4% paraformaldehyde/PBS, stained with 0.5% crystal violet in 25% methanol for 20 min at room temperature, and washed with water. Once the wells had dried, colony formation was document by photography and 10% acetic acid was used to resolubilize crystal violet for spectrophotometry. Absorbance was measured in duplicate at 590 nm. The fraction of surviving colonies was calculated as the ratio of the absorbance of irradiated cells to that of nonirradiated cells. Survival curves were constructed by fitting the average survival levels to a linear quadratic equation using GraphPad Prism.

### Real-Time PCR (RT-PCR)

For RT-PCR, WT, and βarr1^−/−^ MEFs were exposed to IR (4 Gy). RNA was harvested 1 h post IR as well as from untreated control samples, using the Qiagen RNeasy Mini Kit as per manufacturer’s recommendations. Quantification of RNA was performed using the Nanodrop 1000 Spectrophotometer. RNA (2 μg) was reverse transcribed using the High Capacity Reverse Transcription Kit (Applied Biosystems) as per manufacturer’s recommendations. RT-PCR detection with SYBR green was performed with the resulting cDNA and a 50/50 forward primer: reverse primer ratio using the ABI 7900HT Fast Real-Time PCR System. The 53BP1 primer sequences were as follows: 53BP1F—ATTGAACGGTTACCTCAGCCA, 53BP1R—CCCAACTGTGATGAAGCAGAAT. GAPDH primer sequences were as follows: GAPDHF—ACACATTGGGGGTAGGAACA, GAPDHR—AACTTTGGCATTGTGGAAGG. Analysis of 53BP1 as compared with GAPDH was performed using the delta Ct equation [31]. The relative expression of 53BP1 in cells exposed to IR versus untreated controls was determined using the ΔΔCt method [31] and is expressed as arbitrary units.

### Lentiviral and retroviral transduction

Lentiviral particles were produced using HEK293T cells and a third-generation packaging system, MISSION® Lentiviral Packaging Mix, per the manufacturer’s recommendations (Sigma Aldrich). To stably express specific shRNAs, MEF, or U2OS cells were transduced with optimized titers of lentiviruses. Next day medium was changed, and cells were allowed to recover for 24 h before antibiotic selection (2 µg/mL of puromycin) for 3–6 days. A lentiviral CRISPR-Cas9 [32–34] construct containing sgRNA against βarr1 was used to generate U2OS βarr1 KO cell lines, mRNA expression measured by real-time PCR and western blot analysis confirmed deletion of βarr1 expression in the selected clones.

Retroviral transduction was performed with ecotropic viruses. In brief, for the production of virus HEK293T cells were transiently transfected with pcl ECO vector (which expresses the viral genes gag, pol, and envEco) and the vector of interest (1:1 ratio). After 48 h, the supernatant containing the viral particles was diluted 1:2 with fresh medium, filtered, and polybrene was added to a final concentration of 5 µg/mL; MEF cells were transduced with this mix. This procedure was repeated 3 times, every 4 h. Next day medium was changed, and cells allowed to recover for 24 h before antibiotic selection (2 µg/mL of puromycin) for 3–6 days.

### Cycloheximide chase assay

New protein synthesis was blocked by the addition of 25 µg/mL of cycloheximide to tissue culture media. Whole cell lysates were collected at different time points and analyzed by immunoblotting.

### GST pulldown assay

Rat βarr1 was subcloned into pGEX4T1 vector and prepared according to the manufacturer’s recommendations (Amersham Biosciences). 53BP1-myc (2.3 nM), Rad18 (9 nM) and Rad6 (30 nM) were co-incubated with 17 nM of GST-βarr1 or 17 nM of GST at 4 °C overnight in 1 ml binding buffer (50 mM Tris pH = 7.4, 150 mM NaCl, 0.1 mg/mL BSA, 10 μM D-myo-inositol 1,2,3,4,5,6-hexakisphosphate), and then 20 μl of 50% glutathione-sepharose were added to the mixture. The mixture was further incubated at 4 °C for 1 h with rotation. The beads were washed five times with 1 ml binding buffer and separated by SDS-PAGE and analyzed by immunoblotting.

### In-gel trypsin digestion

Immunoprecipitated proteins in the βarr1 signaling complexes were separated by SDS-PAGE (4–20% gradient gel; Invitrogen). The gel lane was excised into six fractions. Each gel fraction was chopped into small pieces and transferred to 1.7 ml Maximum-recovery microcentrifuge tubes and subjected to in-gel trypsin digestion. In brief, the gel pieces were destained by 25 mM ammonium bicarbonate in 50% acetonitrile. The proteins in the gel pieces were reduced by dithiothreitol, alkylated by iodoacetamide, and then subjected to overnight trypsin (working concentration 10 ng/µL) digestion at 37 °C. Tryptic peptides were extracted, lyophilized, resuspended in 40 μL of 5% formic acid, and then further processed on C18 resin, using handmade StageTips. Peptides were eluted with 5% formic acid, 50% acetonitrile, lyophilized with a speed-vac, reconstituted in 0.1% trifluoroacetic acid, 2% acetonitrile, and then subjected to LC-MS/MS analysis.

### Mass spectrometry analysis

LC/MS/MS analyses were performed on a Thermo Scientific LTQ Orbitrap XL (Thermo Scientific) with a Finnigan Nanospray II electrospray ionization source. Peptides were injected onto a 75 μm × 150 mm BEH C18 column (particle size 1.7 μm, Waters) and separated using a Waters nano ACQUITY Ultra Performance LC™ (UPLC™) System (Waters, Milford, MA). The LTQ Orbitrap XL was operated in the data dependent mode using the TOP10 strategy. In brief, each scan cycle was initiated with a full MS scan of high mass accuracy [375–1800 m/z; acquired in the Orbitrap XL at 6 × 104 resolution setting and automatic gain control (AGC) target of 106], which was followed by MS/MS scans (AGC target 5000; threshold 3000) in the linear ion trap on the ten most abundant precursor ions. Selected ions were dynamically excluded for 30 s. Singly charged ions were excluded from MS/MS analysis. MS/MS spectra were searched against a composite database containing the IPI Homo sapiens (human) protein sequences and their reverse sequences using the SEQUEST algorithm. Search parameters allowed two missed tryptic cleavages, a mass tolerance of ±10 ppm for precursor ion, a mass tolerance of ±0.02 D for product ion, a static modification of 57.02146 D (carboxyamidomethylation) on cysteine, and a dynamic modification of 15.99491 D (oxidation) on methionine.

### In vivo study

Wild-type (C57B/6), and *Arrb1*knockout(βarr1^−/−^) mice were exposed to whole-body irradiation (8.75 Gy). Weight loss and overall survival was monitored for 40 days. Animals were euthanized and endpoint apoptosis and DNA damage was assessed in the intestine. All animals used in these studies were adult male mice of 8–12 weeks of age. Animals were handled according to approved protocols and animal welfare regulations of the Institutional Review Board at The Scripps Research Institute.

### Immunohistochemistry

Paraffin sections (3 µm) of intestinal tissue were mounted on Plus slides and dried in a 60 °C oven. The slides were placed on a Leica BondMax Immunostainer and stained with the antibody previously optimized. Slides were dehydrated and cover-slipped with Cytoseal 60 (Richard-Allan Scientific) mounting medium.

### Statistics

Each experiment was repeated at least three times unless indicated otherwise. *P* values were calculated using Student’s *t* test (two-tailed), for survival data, log-rank (Mantel–Cox) test was used (GraphPad, San Diego, CA).

## Results

### βarr1 forms a specific complex with 53BP1

**Fig. 1:**
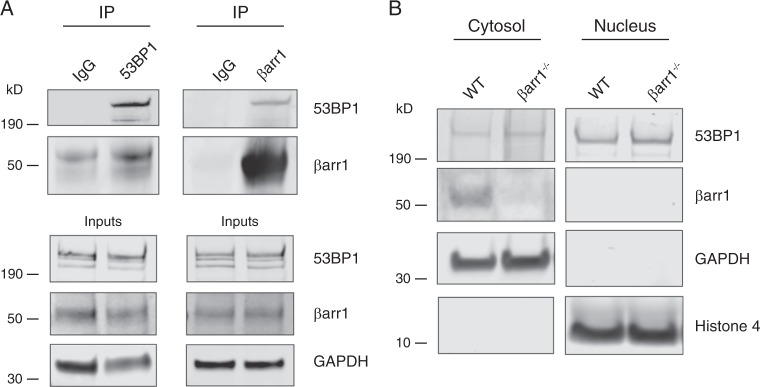
βarr1 binds to 53BP1. **a** Co-immunoprecipitation (co-IP) experiments were performed from cell lysates derived from MEF cells endogenously expressing βarr1 and 53BP1. Clarified lysates were incubated with either, anti-βarr1 (K-16) antibody, anti-53BP1 antibody, or IgG and the resulting products resolved by SDS-PAGE and probed by western blot analysis using antibodies directed against 53BP1 and βarr1. **b** 53BP1 and βarr1 expression in cytosol and nucleus

To define signaling cascades controlled by βarrestins the Lefkowitz laboratory (Duke University, NC) previously conducted global proteomic analyses to identify important binding partners and protein phosphorylation alterations induced specifically by βarrestin signaling [35, 36]. Nucleic acid binding emerged as a large functional category of βarrestin interacting proteins and 53BP1 was identified as a βarr1 interacting partner. To corroborate these results, we performed co-immunoprecipitation experiments from whole cell lysates derived from MEF cells and indeed observed that these proteins interacted or were present within the same complex (Fig. 1a). By sequential isolation of proteins associated with the cytosol, membranes, nucleus, and cytoskeleton from cell lysates, we observed that βarr1 colocalized with 53BP1 in the cytosol (Fig. 1b), which occurs via scaffolding by βarr1 into a multiprotein complex (Supplementary Fig. 1).

### Loss of βarr1 increases 53BP1 foci and radiation resistance

**Fig. 2:**
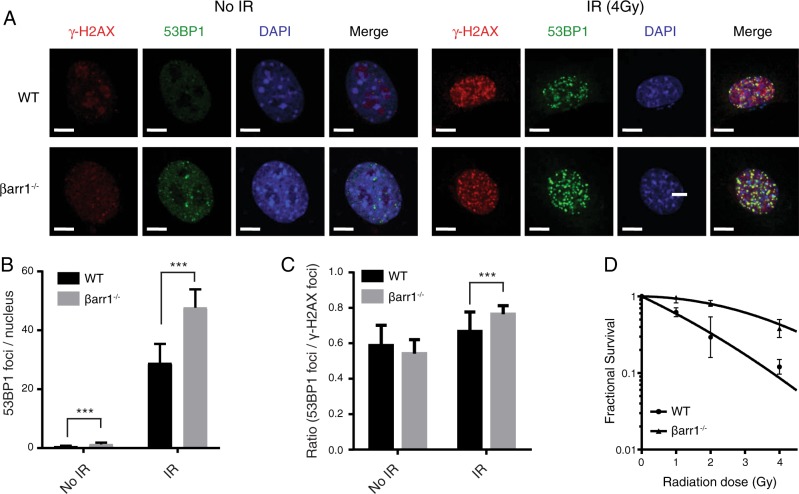
Loss of βarr1 augments 53BP1 foci formation and cell survival following ionizing radiation (IR). **a** Representative confocal immunofluorescence microscopy images of WT and βarr1^−/−^ MEF cells 10 min after 4 Gy IR, probed with anti-53BP1 or anti-γ-H2AX antibodies. Merged image reveals colocalization of 53BP1 foci with γ-H2AX foci and increased 53BP1-containing DNA-repair foci formation in βarr1^−/−^ MEFs compared with WT MEFs in response to IR. Scale bar, 5 µm. **b** Confocal images were used to quantify the number of 53BP1 foci per nucleus in both βarr1^−/−^ and WT MEF cells (***p < 0.0001). **c**, **d** Clonogenic cell survival analysis of WT and βarr1^−/−^ MEFs following exposure to increasing doses of IR. Experiments (*n* = 3) were carried out in triplicate (**p < 0.05 at all doses of IR tested)

Following detection of a DSB, phosphorylation of the histone variant H2AX (γ-H2AX) in chromatin surrounding the break site initiates a cascade of recruiting repair proteins into microscopically visible aggregates known as DNA-repair “foci”. 53BP1 is one member of a large cast of proteins recruited into repair foci upon DNA damage [6]. Accordingly, βarr1 knockout (βarr1^−/−^) MEF cells display an increase in 53BP1 foci size and intensity that results in a significant increase in overlap with γ-H2AX foci in βarr1^−/−^ cells compared with WT cells (Fig. 2a). This observation correlates with significantly increased numbers of 53BP1 foci compared with paired wild type (WT) in response to DNA damage induced by IR (Fig. 2b, c). Importantly, this increase in 53BP1 repair foci observed in βarr1^−/−^ MEFs translates into a remarkably enhanced cell survival following exposure to irradiation (Fig. 2d, Supplementary Table 1), suggesting that loss of βarr1 leads to an increased capacity to repair or tolerate damaged DNA.

### 53BP1 protein levels correlate with cell survival after irradiation

**Fig. 3:**
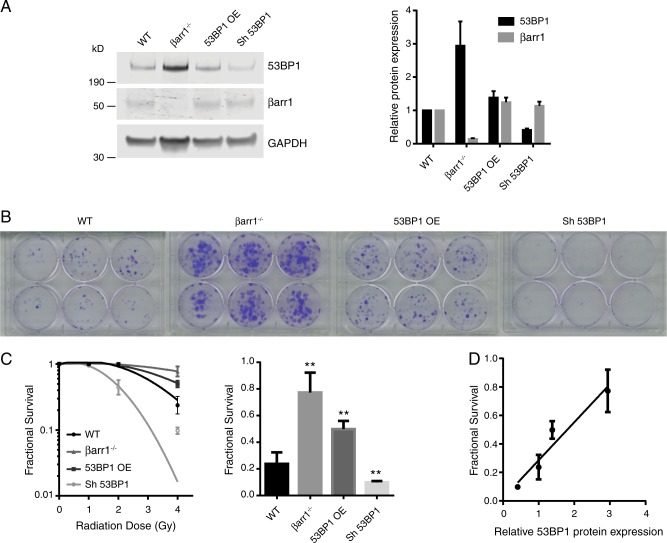
53BP1 protein expression directly correlates with cell survival. **a** 53BP1 was either overexpressed or downregulated in MEF cells obtaining a range of different expression levels of the protein. Data are mean ± s.e.m of three independent experiments. **b** The clonogenicity assay images corresponding to 4 Gy irradiation. **c** Clonogenic cell survival analysis in MEF cells revealed a significant difference in survival after exposure to IR in the conditions tested; bar graph for 4 Gy (**p < 0.01 compare with WT). Data are mean ± s.e.m of an experiment run *n* = 6. **d** Fractional survival plot as a function of 53BP1 protein expression levels (*r* = 0.85)

To test whether 53BP1 is required and responsible for the increased survival of βarr1^−/−^ cells after exposure to IR (4 Gy), we overexpressed (53BP1 OE) or downregulated (Sh 53BP1) 53BP1 in WT MEFs (Fig. 3a). Notably, the 53BP1 protein levels in the 53BP1 OE MEFs were 1.5-fold versus WT (not as high as those observed in βarr1^−/−^ MEFs), significantly improving the survival rate after IR (4 Gy) compared with that of WT MEFs (Fig. 3b, c). Conversely, knockdown of 53BP1 increased the sensitivity of MEFs to IR (Fig. 3b, c). Importantly, these data support the hypothesis that the βarr1-53BP1 signaling axis plays a key role in the observed IR tolerance. Furthermore, we observed a direct correlation between 53BP1 protein levels and resistance of MEFs (Fig. 3d),

### Regulation of 53BP1 by βarr1 occurs post translationally

**Fig. 4:**
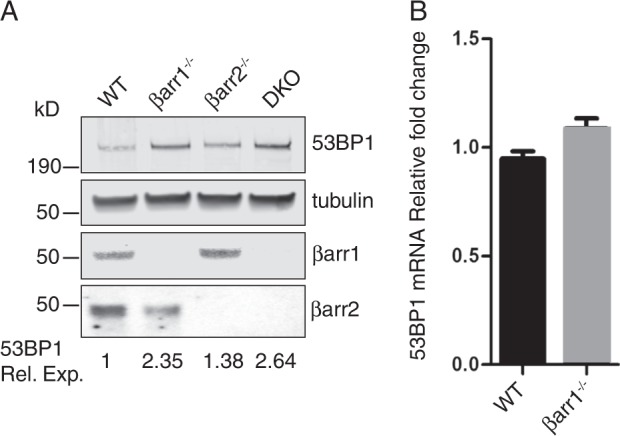
βarr1 post transcriptionally regulates expression levels of 53BP1. **a** Cell lysates derived from WT, βarr1^−/−^, βarr2^−/−^ MEFs, and βarr1^−/−^; βarr2^−/−^ double knockout MEFs (DKO) were immunoblotted and analyzed using the indicated antibodies. **b** Real-time PCR analysis of 53BP1 mRNA levels in paired WT and βarr1^−/−^ MEFs following exposure to IR

To gain mechanistic insight into the radiation resistance associated with βarr1 silencing, we examined 53BP1 expression levels in WT, βarr1^−/−^, βarr2^−/−^, and double knockout (DKO: βarr1^−/−^ βarr2^−/−^) MEFs. Both βarr1^−/−^ and DKO MEFs had increased levels of 53BP1 protein compared with the WT and βarr2^−/−^ MEFs (Fig. 4a), indicating a potential role of βarr1 in regulating 53BP1. Interestingly, Kim et al. demonstrated that activation of the nuclear factor-erythroid 2-related factor 2 by the synthetic triterpenoid bardoxolone methyl, upregulates 53BP1 gene expression and acts as an IR protectant [37]. To evaluate whether regulation of 53BP1 by βarr1 is pre- or post translational, the levels of 53BP1 mRNA in WT and βarr1^−/−^ MEFs were assessed by qRT-PCR. There were no significant differences in the levels of 53BP1 transcripts between WT and βarr1^−/−^ MEFs (Fig. 4b), suggesting that βarr1 plays a posttranscriptional role.

### 53BP1 protein levels are dependent on βarr1

**Fig. 5:**
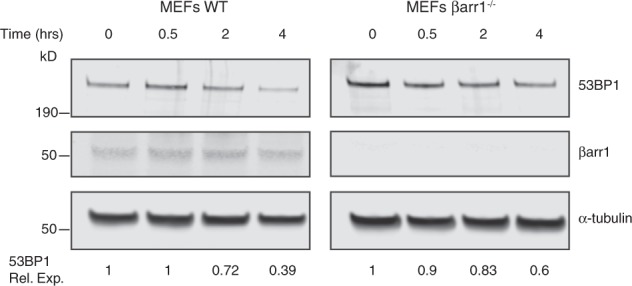
Turnover of 53BP1 is dependent on βarr1. Lysates derived from WT and βarr1^−/−^ MEF cells treated with 25 µg/ml of cycloheximide, were subjected to western blot analysis and analyzed for 53BP1 levels. Rel. Exp. relative expression

In order to discern if the accumulation of 53BP1 that is observed in the βarr1^−/−^ MEF cells is due to improved stability of 53BP1 or an altered steady state rate of turnover, we blocked new protein synthesis by the addition of cycloheximide [38]. Comparison of the 53BP1 levels over time, between the WT and βarr1^−/−^ MEF cells post cycloheximide addition, reveals that 53BP1 protein levels have a slower rate of turnover in βarr1^−/−^ MEFs cells (Fig. 5). This is consistent with the proposal that βarr1 controls 53BP1 degradation.

### βarr1 regulates DNA repair by acting as an E3-ubiquitin ligase adaptor for 53BP1 through a complex with Rad18/Rad6

**Fig. 6:**
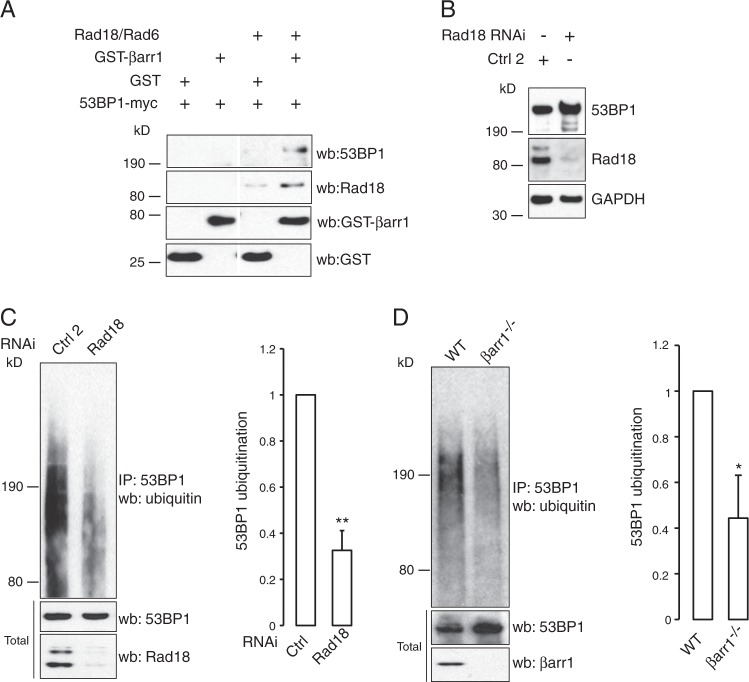
βarr1 forms a complex with Rad18/Rad6 promoting polyubiquitylation of 53BP1. **a** Immunoblot showing complex formation of βarr1, Rad18/Rad6, and 53BP1 in vitro. Purified 53BP1-myc was incubated with either GST or GST-βarr1 in the presence or absence of Rad18/Rad6. Proteins residing in the precipitates were subjected to western blot analysis and probed with the indicated antibodies. Presented data was cropped from the same blot image exposed for an equal time-period. **b** HEK293 cells were transfected with either control (Ctrl 2) siRNA or siRNA directed against Rad18 and 53BP1 levels were determined by immunoblotting. **c** HEK293 cells were transfected with either control (Ctrl) siRNA or siRNA directed against Rad18 and treated with 2 µg/mL bleomycin (BLM). Cell lysates were immunoprecipitated with anti-53BP1 antibody and levels of polyubiquitylated 53BP1 were assessed by western blot using an anti-ubiquitin (P4D1) antibody. All bars represent mean ± s.e.m of three independent experiments. **d** Lysates derived from WT and βarr1^−/−^ MEFs treated with 2 µg/mL BLM were immunoprecipitated using an anti-53BP1 antibody, immunoblotted, and probed with an anti-ubiquitin (P4D1) antibody. All bars represent mean ± s.e.m. of three independent experiments

βarr1 is known to function as an E3-ubiquitin ligase adaptor [39], which we have demonstrated influences genomic stability by facilitating MDM2-dependent p53 degradation in response to stress induced by chronic β2AR signaling [29]. Mass spectrometry of proteins that bind to βarr1 in cells in response to IR revealed that the E3-ubiquitin ligase Rad18, which has previously been shown to mono-ubiquitylate 53BP1 and lead to its retention with chromatin [40–44], associates with βarr1 (Supplementary Fig. 1). Subsequent in vitro binding studies with purified recombinant proteins demonstrated that 53BP1 and βarr1 do not directly associate, but rather require the presence of Rad18 and its E2-ubiquitin-conjugating enzyme Rad6 (Fig. 6a). Thus, βarr1 may form a ternary complex comprised of the E3/E2-ubiquitin ligase Rad18/Rad6 heterodimer and 53BP1.

To test if Rad18 influences 53BP1 protein levels in cells, we performed knockdown studies using Rad18-specific siRNAs. Efficient knockdown of Rad18 led to an increase in the level of 53BP1 protein (Fig. 6b); indicating that Rad18 is an important regulator of steady state levels of 53BP1. Moreover, Rad18 knockdown decreased levels of polyubiquitylated 53BP1 (Fig. 6c). Finally, consistent with the notion that βarr1 facilitates Rad18/53BP1 interaction, βarr1^−/−^ MEFs had reduced levels of polyubiquitylated 53BP1 compared with WT MEFs (Fig. 6d). Collectively, these findings support a role for βarr1 as an E3-ubiquitin ligase adaptor for 53BP1.

### βarr1 deficiency increases survival after irradiation in vivo

**Fig. 7:**
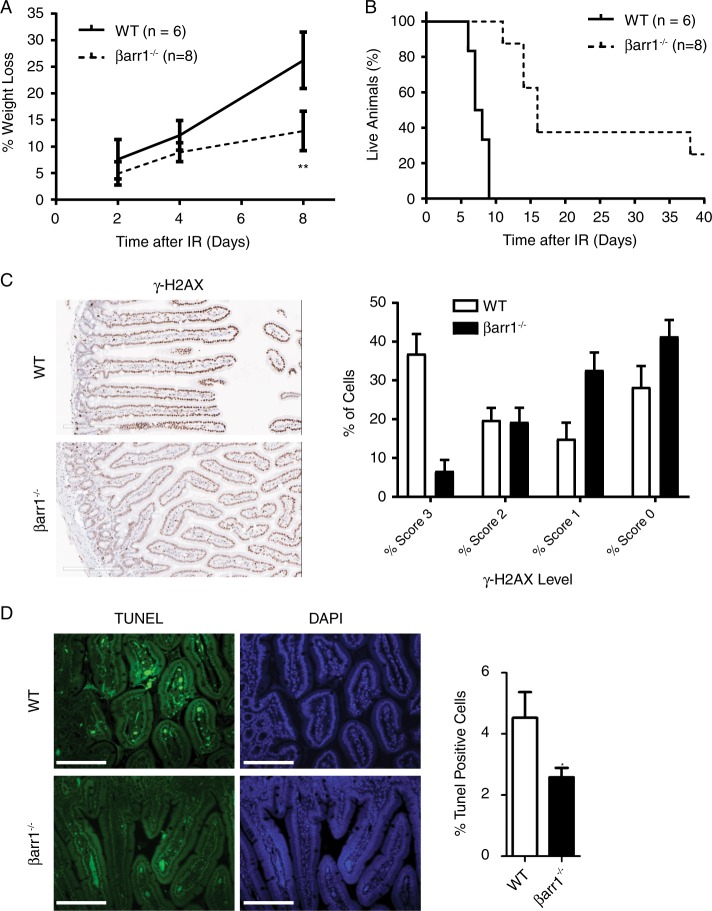
βarr1 deficiency augments survival and reduces intestinal injury in mice exposed to lethal doses of IR. **a** Weight loss in βarr1^−/−^ mice is decreased as compared with WT mice in response to 8.75 Gy total-body irradiation (TBI) (**p < 0.001). **b** Increased survival rate of βarr1^−/−^ mice in response to 8.75 Gy TBI (***p < 0.0001). **c** IR-induced γ-H2AX staining showed lower intensity in tissue from βarr1^−/−^ mice as compared with WT. Pathological scores were given using Aperio Image Scope software. **d** TUNEL analysis showing reduced apoptosis in the intestinal crypts of βarr1^−/−^ mice as compared with WT mice 5 h after 8.75 Gy TBI. Scale bar, 200 µm. High-content image quantification of TUNEL staining in WT and βarr1^−/−^ crypt cells. (*p < 0.05)

To test if βarr1-dependent control of 53BP1 influences the DDR in vivo, WT and βarr1^−/−^ mice were exposed to whole-body irradiation (8.75 Gy); levels that are sufficient to model acute radiation syndrome (ARS) [45]. Six days post IR, WT mice exhibited augmented weight loss (8–16%) versus βarr1^−/−^ mice (6–11%), and by 8 days post IR weight loss of βarr1^−/−^ mice (average of 13%) was half of that of WT mice (26%, Fig. 7a). This was followed by deaths of the WT mice as early as 6 days after exposure, and all WT animals were dead by day 9 (Fig. 7b). In contrast, all the βarr1^−/−^ mice were still alive at day 9 (Fig. 7b). Thus, the βarr1 deficiency is associated with radiation resistance in vivo.

ARS is associated with severe toxicity and failure of the hematopoietic, gastrointestinal, and cerebrovascular systems [45]. Since we observed significant weight loss preceding the IR-induced death of WT mice, we examined apoptosis in intestinal crypt cells, which are one of the most highly replicative cell types and are known to be exquisitely sensitive to the acute effects of IR [45]. Consistent with the IR resistant phenotype of βarr1^−/−^ mice, there were significant reductions in DNA damage as shown by lower intensity of γ-H2AX staining (Fig. 7c). This correlated with a reduction in apoptosis in the intestinal crypt cells of IR-treated βarr1^−/−^ mice compared with IR-treated WT mice (Fig. 7d). These findings are consistent with the notion that increased levels of 53BP1 that are manifest in βarr1-deficient cells augment DNA repair in IR-treated βarr1^−/−^ mice.

### βarr1 forms a complex with 53BP1 in human cells

**Fig. 8:**
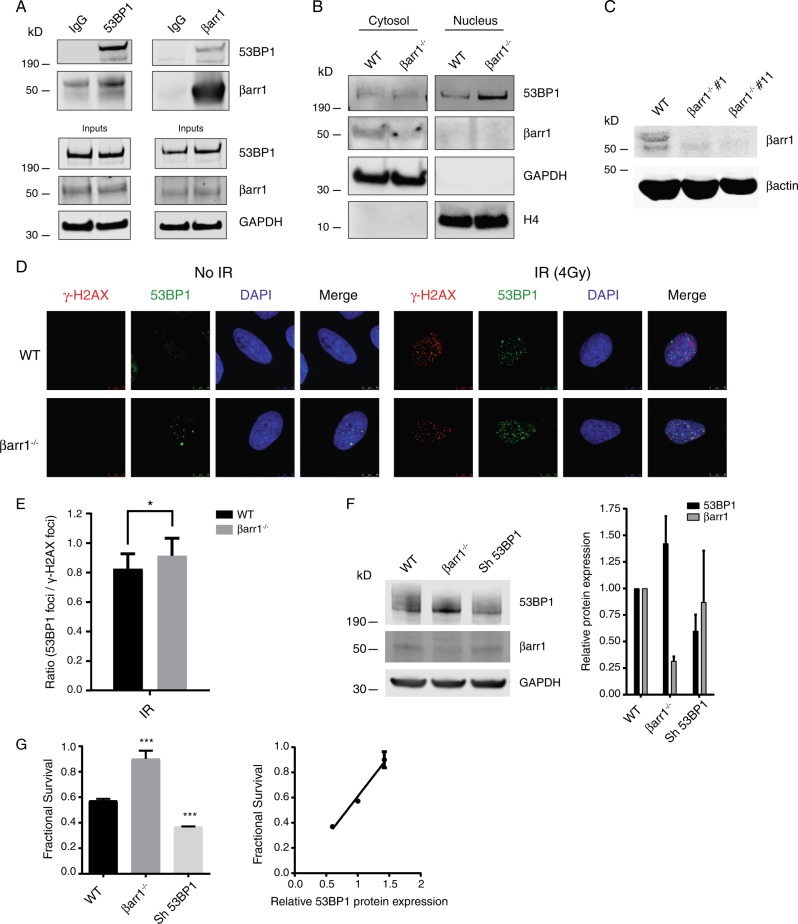
βarr1 forms a complex with 53BP1 and influences the DNA damage response also in human cells. **a** Co-immunoprecipitation (co-IP) experiments were performed from cell lysates derived from U2OS cells endogenously expressing βarr1 and 53BP1. Clarified lysates were incubated with either anti-βarr1 (K-16) antibody, or anti-53BP1 antibody or IgG and the resulting products resolved by SDS-PAGE and probed by western blot analysis using antibodies directed against 53BP1 and βarr1. **b** 53BP1 and βarr1 expression in cytosol and nucleus. **c** Cell lysates derived from WT U2OS, U2OS/Cas9/ARRB1-gRNA1 clone 1 (βarr1^−/−^ #1) and U2OS/Cas9/ARRB1-gRNA1 clone 11 (βarr1^−/−^ #11) were immunoblotted and analyzed using the indicated antibodies. βarr1^−/−^ #11 was selected for further assays. **d** Representative confocal immunofluorescence microscopy images of WT and βarr1^−/−^ U2OS cells 10 min after 4 Gy IR, probed with anti-53BP1 or anti-γ-H2AX antibodies. Merged image reveals colocalization of 53BP1 foci with γ-H2AX foci and increased 53BP1-containing DNA-repair foci formation in βarr1^−/−^ U2OS compared with WT U2OS in response to IR. **e** High-content imaging quantification comparing the ratio of 53BP1 foci to γ-H2AX foci in both βarr1^−/−^ and WT U2OS cells (*p < 0.05). **f** βarr1 and 53BP1 were downregulated in U2OS cells obtaining a range of different expression levels of 53BP1. Data are mean ± s.e.m of three independent experiments. **g** Clonogenic cell survival analysis in U2OS cells revealed a significant difference in survival after exposure to IR in the conditions tested; bar graph for 4 Gy (***p < 0.0001 compare with WT). Data are mean ± s.e.m of an experiment run *n* = 3. Fractional survival plot as a function of 53BP1 protein expression levels (*r* = 0.93) in U2OS cells

Co-immunoprecipitation experiments performed using cell lysates from human osteosarcoma (U2OS) cells demonstrate that 53BP1 is also an interacting partner of βarr1 in human cells (Fig. 8a), where both proteins colocalize in the cytosol (Fig. 8b). CRISPR/ Cas9 technology was used to knockout βarr1 in U2OS cells (Fig. 8c). Immunofluorescence studies show increased 53BP1 foci (Fig. 8d, e) after irradiation in βarr1^−/−^ cells compare with WT cells. Moreover, βarr1^−/−^ cells showed a significant increase of 53BP1 protein levels (Fig. 8f), which correlates with improved survival after IR (4 Gy) compared with that of WT U2OS cells (Fig. 8g), suggesting that post-transcriptional regulation of 53BP1 by βarr1 may be a general response. While further studies are needed, the data suggest the βarr1 may also play an upstream role in the DDR in human cells.

## Discussion

The diverse signaling functions of βarr1 were recently revealed using a global mass spectrometry approach, which identified numerous βarr1 binding partners [35]. Included within the cast of proteins that βarr1 interacts with are those with known functions in DNA repair. We have previously demonstrated that βarr1 controls p53 levels in response to catecholamine-induced chronic stress, by acting as a scaffold for the E3-ubiquitin ligase MDM2 that targets p53 for degradation. This stress-induced genomic instability is βarr1-mediated, as genetic deletion of βarr1 dramatically reduces DNA damage in vivo in response to treatment with the β2-adrenergic receptor agonist, isoproterenol [29]. Further, βarr1^−/−^ mice subjected to 2 weeks of chronic restraint had significantly less DNA damage in the frontal cortex compared with their wild-type counterparts [46]. Here we establish an additional role for βarr1 in the response to IR, where we show βarr1 also controls the destruction of the DNA-repair protein 53BP1.

Mass spectrometry and immunoprecipitation experiments demonstrate that βarr1 functions as an E3-ubiquitin ligase adaptor for 53BP1, promoting ubiquitination and degradation of 53BP1 by the E3-ubiquitin ligase Rad18 in complex with the E2-ubiquitin-conjugating enzyme Rad6. The interaction between βarr1 and 53BP1 is indirect, where it appears that βarr1 scaffolds the Rad18/Rad6 heterodimer, which then binds to and polyubiquitylates 53BP1 (Fig. 6). Rad18 plays an important role in several DDR processes, where it monoubiquitylates proliferating cell nuclear antigen at replication forks that are stalled by UV-induced DNA lesions [47] and facilitates chromatin retention of 53BP1 in response to IR-induced DSB [40]. In our studies, immunofluorescence analyses demonstrate that formation of the βarr1-Rad18-Rad6-53BP1 ternary complex is not observed within the DSB-repair foci (data not shown). Thus, we hypothesize that this complex maintains steady state levels of 53BP1, whereby silencing βarr1 or Rad18 raises 53BP1 levels which would be available to promote 53BP1 IR-induced DNA-repair foci formation. Further, the increase in the number and duration of 53BP1-containing foci correlates with in vitro cellular resistance to IR with a significant dose modifying factor observed at each irradiation dose tested (Supplementary Table 1). This is highlighted by the correlation comparing 53BP1 protein levels and the resistance of MEF cells with IR (Fig. 3). Reduction of 53BP1 partially increases the sensitivity to IR while conversely, overexpression of this protein provides protection against it. These data suggest that βarr1 plays an important role in DNA repair in mice through both modulating p53 [29] as well as post translational control of 53BP1 levels. Moreover, this observations translates in vivo, were βarr1 deficiency promotes prolonged survival after whole-organism irradiation, consistent with reduced IR-induced γ-H2AX staining and apoptosis in these mice (Fig. 7).

Two major pathways of DSB repair are HR and NHEJ. HR requires a homologous DNA template and the process of gene conversion is a high-fidelity process that occurs during late S and G2 phases of the cell cycle. By contrast, NHEJ involves the direct ligation of the break, and depending upon the extent of processing at the break site, the process of NHEJ may have a higher probability for reduced fidelity. DNA DSBs are repaired via both fast and slow kinetics in chromatin, and the majority of IR-induced repair in mammalian cells occurs via the more efficient NHEJ pathway [48, 49]. The utilization of NHEJ may be particularly necessary when a highly proliferative cell suffers an excessive number of DSBs as occurs following IR exposure [50].

We have demonstrated that silencing βarr1 protects against IR-induced DNA damage in both mouse and human cells, although the magnitude of the observed response seems to be larger in mouse cells, likely reflecting the multilevel control of 53BP1 regulation in human cells. Since 53BP1 protein levels and chromatin binding differ in mouse and human systems, control of 53BP1 protein levels by βarr1-Rad18 might be more important in mouse than human cells. For example, the 53BP1 binding partner TIRR (Tudor-interacting repair regulator) regulates DNA repair by sequestering 53BP1 and preventing its loading onto chromosomes [20]. Moreover, 53BP1 is recruited to DNA double-strand breaks by histone marks including those catalyzed and regulated by the RNF8/RNF168/TRIP12/UBR5 signaling complex [51]. Further studies will be required to determine whether βarr1 alters the dynamics and activities of proteins required for 53BP1 chromatin loading.

Our ex vivo studies demonstrate that silencing βarr1 substantially protects cells from IR-induced toxicity. As mentioned before, this protection translates in vivo and may also account for the reduced GI toxicity observed during IR-induced ARS in βarr1^−/−^ mice (Fig. 7). These studies suggest that pharmacological blockade of the βarr1-53BP1 signaling cascade provides a novel strategy for developing therapeutic agents with radiation protection properties through enhanced repair of IR-induced DNA DSBs. There are several radiation countermeasures under clinical development which are supported by the Department of Defense and the Armed Forces Radiation Research Institute [52]. While each approach is promising, the mechanisms of action of the current agents act upstream of the IR-induced DNA break itself. We anticipate that pharmacologically mimicking the βarr1 knockout phenotype would act as an effective countermeasure to IR and may also augment the efficacy of the current molecules under investigation.

BRCA1 is an essential component of HR-mediated repair, and tumors arising in patients with mutated BRCA1 have evidence of genomic instability and defects in DNA repair [53–55]. Indeed, this repair defect has been exploited to develop “synthetic lethal” treatment strategies using PARP inhibitors. However, the efficacy of PARP inhibitors has been plagued with resistance mechanisms that restore the repair capacity of HR, including secondary mutations in BRCA1, loss of 53BP1, or silencing of the subunits compromising the multiprotein Shieldin complex which can reverse many aspects of the BRCA1 deficient phenotype [16–18, 56]. Mechanistically, studies indicate that Shieldin, like 53BP1, blocks DNA end resection, thus playing a role in directing the choice of pathway for DSB repair [11–14, 19]. Our current studies demonstrating that βarr1 controls the levels of 53BP1 suggest that this circuit may play roles in tumor initiation and therapy resistance of BRCA1 deficient breast tumors. Importantly, they also suggest that the βarr1-53BP1 circuit could be targeted for sensitizing certain breast cancers to PARP inhibitors, as well as protect patients from the effects of high doses of IR.

## Supplementary information

Supplementary Materials Legends

Supplementary Figure 1

Supplementary Table 1
