# Supplementary material for: βarrestin-1 regulates DNA repair by acting as an E3-ubiquitin ligase adaptor for 53BP1
Source: Cell Death Differ. 2019 Sep 10;27(4):1200–13. doi: 10.1038/s41418-019-0406-6 (PMC7206116; doi:10.1038/s41418-019-0406-6)
Supplement: Supplementary file 1 — Supplementary Materials Legends [file 41418_2019_406_MOESM1_ESM.docx]

**SUPPLEMENTARY MATERIALS**

**Supp. Fig. 1. Identification of Rad18 as a βarr1 binding partner during DNA damage by mass spectrometry. (A)** Rad18, an E3 ubiquitin ligase, was identified by mass spectrometry analysis in the βarr1 signaling complexes pulled-down from HEK293 cells stably over-expressing Flag-tagged βarr1 under gamma radiation condition. The sequences of the two peptides (58TQCPTCCVTVTEPDLK73 and 174SVEEIAPDPSEAK186) identified were listed. The mass spectrometry analysis was performed using a Thermo scientific LTQ Orbitrap XL mass spectrometer. Rad18 peptides were not detected in the control samples without gamma radiation condition. **(B)** and **(C)** show the annotated MS/MS fragmentation spectra for Rad18 peptides 58TQCPTCCVTVTEPDLK73 and 174SVEEIAPDPSEAK186, respectively. The peptide sequences are shown at the top of the MS/MS spectra. The identified fragmentation y (red color) and b (blue color) ions are indicated.

**Supp. Table 1. Quantification of survival fraction and calculation of dose modifying factor (DMF).**
